# Prevalence and novelty of *PRPF31 *mutations in French autosomal dominant rod-cone dystrophy patients and a review of published reports

**DOI:** 10.1186/1471-2350-11-145

**Published:** 2010-10-12

**Authors:** Isabelle Audo, Kinga Bujakowska, Saddek Mohand-Saïd, Marie-Elise Lancelot, Veselina Moskova-Doumanova, Naushin H Waseem, Aline Antonio, José-Alain Sahel, Shomi S Bhattacharya, Christina Zeitz

**Affiliations:** 1 INSERM, UMRS968, Paris, F-75012, France; 2 UPMC Univ Paris 06, UMR_S 968, Institut de la Vision, Paris, F-75012, France; 3 CNRS, UMR_7210, Paris, F-75012, France; 4 Centre Hospitalier National d'Ophtalmologie des Quinze-Vingts, INSERM-DHOS CIC 503, Paris, F-75012, France; 5 UCL-Institute of Ophthalmology, Bath Street, London, UK

## Abstract

**Background:**

Rod-cone dystrophies are heterogeneous group of inherited retinal disorders both clinically and genetically characterized by photoreceptor degeneration. The mode of inheritance can be autosomal dominant, autosomal recessive or X-linked. The purpose of this study was to identify mutations in one of the genes, *PRPF31*, in French patients with autosomal dominant RP, to perform genotype-phenotype correlations of those patients, to determine the prevalence of *PRPF31 *mutations in this cohort and to review previously identified *PRPF31 *mutations from other cohorts.

**Methods:**

Detailed phenotypic characterization was performed including precise family history, best corrected visual acuity using the ETDRS chart, slit lamp examination, kinetic and static perimetry, full field and multifocal ERG, fundus autofluorescence imaging and optic coherence tomography. For genetic diagnosis, genomic DNA of ninety families was isolated by standard methods. The coding exons and flanking intronic regions of *PRPF31 *were PCR amplified, purified and sequenced in the index patient.

**Results:**

We showed for the first time that 6.7% cases of a French adRP cohort have a *PRPF31 *mutation. We identified in total six mutations, which were all novel and not detected in ethnically matched controls. The mutation spectrum from our cohort comprises frameshift and splice site mutations. Co-segregation analysis in available family members revealed that each index patient and all affected family members showed a heterozygous mutation. In five families incomplete penetrance was observed. Most patients showed classical signs of RP with relatively preserved central vision and visual field.

**Conclusion:**

Our studies extended the mutation spectrum of *PRPF31 *and as previously reported in other populations, it is a major cause of adRP in France.

## Background

Rod-cone dystrophies, also called retinitis pigmentosa (RP), are a clinically and genetically heterogeneous group of inherited retinal disorders usually primarily affecting rods with secondary cone degeneration [1-4]. It represents a progressive disorder which often starts with night blindness and leads to visual field constriction, abnormal color vision and can eventually lead to loss of central vision and complete blindness. It is the most common inherited form of severe retinal degeneration, with a frequency of about 1 in 4000 births and more than 1 million individuals affected worldwide. The mode of inheritance can be X-linked (5-15%), autosomal dominant (30-40%) or autosomal recessive (50-60%). The remaining patients represent isolated cases for which the inheritance trait cannot be established [5].

To date, mutations in 20 different genes are associated with autosomal dominant RP (adRP) http://www.sph.uth.tmc.edu/Retnet/. The majority of prevalence studies reveal rhodopsin (*RHO*) being the most frequently mutated gene in adRP [6]. *PRPF31 *was also proposed to represent a major gene underlying this disorder. It is located on chromosome 19q13.42, encompasses 14 exons and codes for a ubiquitously expressed pre-mRNA splicing factor [7]. According to published reports, *PRPF31 *mutation prevalence ranges from 1 to 8% in adRP cohorts from various geographical origins, with higher frequencies reported in the United States [8-15].

To date over 40 mutations have been located in different parts of the gene. The mutation spectrum comprises missense, splicing, regulatory and nonsense mutations. In addition small or gross insertions, small insertion-deletions, and small or gross deletions were identified (http://www.sph.uth.tmc.edu/Retnet/, http://www.retina-international.org/sci-news/prp31mut.htm) (Table 1).

**Table 1:** Previously described *PRPF31 *mutations in adRP patients.

Exon/Intron	Nucleotide Exchange	Protein Effect	Publication	Information about penetrance
Int1	c.1-2481G>T (formerly: IVS1+1G>T)	splice defect	[27]	incomplete

2	c.79G>T	p.Glu27X	[15]	Incomplete

Int2	c.177+1G>A	splice defect	[13] [23]	Simplex Simplex

3	c.220C>T	p.Gln74X2	[13]	Simplex

4	c.319C>G	p.Leu107Val (interferes with splice site leading to frameshift)	[23]	Simplex

Int4	c.323-2A>G	Splice defect	[23]	Simplex

5	c.331_342del	p.His111_Ile114del	[22]	High

5	c.358_359delAA	p.Lys120GlufsX122	[9]	Simplex

5	c.390delC	p.Asn131MetfsX67 (formerly p.Asn131fs7ter197)	[13]	segregates in 3 affected

5	c.413C>A (formerly c.412C>A)	p.Thr138Lys	[15]	Incomplete

Int5	c.421-1G>A	splice defect	[28]	Incomplete

6	c.421G>T	p.Glu141X	[13]	Simplex

Int6	c.527+1G>T	splice defect	[29]	Incomplete

Int6	c.527+1G>A	splice defect	[9]	Incomplete

Int6	c.527+3A>G	splice defect	[7,16] [15]	Incomplete incomplete

Int6	c.528-1G>A	splice defect	[15]	not tested

Int6	c.528-3_45del (previous description: IVS6-3 to -45 del)	splice defect	[7,12]	Incomplete

7	c.581C>A	p.Ala194Glu	[7]	Simplex

7	Formerly: 580-581dup33bp	formerly: in frame insertion of 11 amino acids	[7]	Simplex

7	c.636delG	p.Met212IlefsX27 (formerly: Met212fs/ter238)	[13]	segregates in 2 affected

7	c.646G>C	p.Ala216Pro	[7]	Incomplete

8	c.732_737delins20bp	p.Met244fsX248	[11]	segregated in 2 affected

8	c.758_767del	p.Gly253AlafsX65 (formerly: p.Gly253fs/ter317)	[13]	Simplex

8	c.769_770insA	p.Thr258AspfsX21 (formerly: frameshift, 20 novel amino acids then STOP) (formerly:Lys257fsX277)	[7] [11]	Simplex incomplete

8	c.785delT	p.Phe262SerfsX59	[10]	

8	c.828_829delCA	p.His276GlnfsX2 (formerly p.His276fsX237)	[11]	Incomplete

Int8	c.856-2A>G	splice defect	[23]	segregated in 2 affected

9	c.871G>C	p.Ala291Pro	[13]	Simplex

9	c.877_910del	p.Arg293_Arg304>ValfsX17	[23]	Incomplete

9	c.895T>C	p.Cys299Arg	[13]	Incomplete

10	c.973G>T	p.Glu325X	[13]	Simplex

10/int10	1049_IVS10+20del/insCCCCT	splice defect	[13]	Simplex

Int10	c.1073+1G>A (Formerly:IVS10+1G>A)	splice defect	[13]	segregates in 5 affected

11	c.1115_1125del	p.Arg372GlnfsX99 (formerly: frameshift, 98 novel amino acids then STOP)	[7]	Incomplete

11	c.1142delG	p.Gly381GlufsX32	[12]	Incomplete

Int11	c.1146+2T>C	Splice defect	[15]	Incomplete

12	c.1155_1159delGGACG/insAGGGATT	p.Asp386GlyfsX28	[12]	Incomplete

Int13	c.1374+654C>G	Splice defect	[19]	Incomplete

ex1/int1	indel ex1/int1	Loss of one copy of *PRPF31*	[14]	Incomplete

4-8	4.8 kb deletion	Loss of one copy of *PRPF31*	[14]	simplex

4-13	11.3 kb deletion	Loss of one copy of *PRPF31*	[14]	incomplete

*PRPF31: 1-11, TFPT*, *NDUFA3*, partly *OSCAR*	59 kb deletion	Loss of one copy of *PRPF31*	[30]	incomplete

*PRPF31*, *TFPT*, *NDUFA3*, partly *OSCAR*	32-42 kb deletion	Loss of one copy of *PRPF31*	[14]	simplex

*PRPF31*, *TFPT*, *NDUFA3, OSCAR*	> 44.8 kb deletion	Loss of one copy of *PRPF31*	[14]	simplex

*PRPF31 *without Stop codon, *TFPT NDUFA3*, promoter *OSCAR*	30 kb deletion	Loss of one copy of *PRPF31*	[31]	incomplete

If possible, mutations are indicated according to NM_015629 by using the recommendations of human genome variation society: http://www.hgvs.org/rec.html and/or the nomenclature of the original publication are given

The phenotype, age of onset and the severity of the disease in adRP patients varied with different *PRPF31 *mutations. In addition, in some families the same mutation was even associated with a range of phenotypic variations [15]. Furthermore, several studies revealed that incomplete penetrance is a common feature in families showing *PRPF31 *mutations with an asymptomatic mutation carrier having a carrier child, who fully manifests the disease [16-18].

Our comprehensive study reported here aims to perform for the first time genotype-phenotype correlations in a French adRP cohort with *PRPF31 *mutations. All patients were recruited from the same clinical center, namely the Quinze-Vingts hospital in Paris. We will present the prevalence of *PRPF31 *mutations in this cohort and compare our findings with other studies.

## Methods

### Clinical assessment

Ninety families with a provisional diagnosis of autosomal dominant rod-cone dystrophy, (adRP) were ascertained in the Clinical Investigating Centre of Quinze-Vingts Hospital. Informed consent was obtained from each patient and normal controls after explanation of the study and its potential outcome. The study protocol adhered to the tenets of the Declaration of Helsinki and was approved by the local ethics committee. Each patient underwent full ophthalmic examination with clinical assessment as described earlier [6]. For additional family members who could not come to our centre for examination, ophthalmic records were obtained from local ophthalmologists.

### Mutation detection

Total genomic DNA was extracted from peripheral blood leucocytes according to manufacturer recommendation (Puregen Kit, Qiagen, Courtaboeuf, France). Subsequently, direct genomic sequencing of *PRPF31 *was performed. All 14 exons of which exons 2-14 are coding, and flanking intronic regions of *PRPF31 *were PCR amplified in 10 fragments (*PRPF31 *RefSeq NM_015629) using oligonucleotides previously described [7] and a polymerase (HotFire, Solis Biodyne, Estonia) in the presence of 2.5 mM MgCl_2 _and at an annealing temperature of 60°C. The PCR products were enzymatically purified (ExoSAP-IT, USB Corporation, Cleveland, Ohio, USA purchased from GE Healthcare, Orsay, France) and sequenced with a commercially available sequencing mix (BigDyeTerm v1.1 CycleSeq kit, Applied Biosystems, Courtaboeuf, France). The sequenced products were purified on a presoaked Sephadex G-50 (GE Healthcare) 96-well multiscreen filter plate (Millipore, Molsheim, France), the purified product analyzed on an automated 48-capillary sequencer (ABI 3730 Genetic analyzer, Applied Biosystems) and the results interpreted by applying a software (SeqScape, Applied Biosystems). At least 192 commercially available control samples were used to validate the pathogenicity of the novel sequence variants (Human random control panel 1-3, Health Protection Agency Culture Collections, Salisbury, United Kingdom).

Multiplex Ligation dependent Probe Amplification (MLPA) was performed using a commercially available kit (SALSA MLPA kit P235-B1 Retinitis, MRC Holland). The MLPA reactions were carried out according to the manufacturer's instructions and analyzed on an automated 48-capillary sequencer (ABI 3730 Genetic analyzer, Applied Biosystems). MLPA data analysis was performed using GeneMarker (Softgenetics) and additionally Coffalyser (MRC Holland) software. Five control DNAs were included in each MLPA run and the data was interpreted in terms of the ratio of each probe signal between the control and patient DNA samples. Samples with probe ratio values below 0.6 were considered as deletions and values above 1.4 as duplications.

## Results and Discussion

Samples included in this study are part of a French cohort of adRP patients that were previously screened for *RHO *mutations and we noted 16.5% of cases with known or novel *RHO *mutations [6].

**Figure 1:**
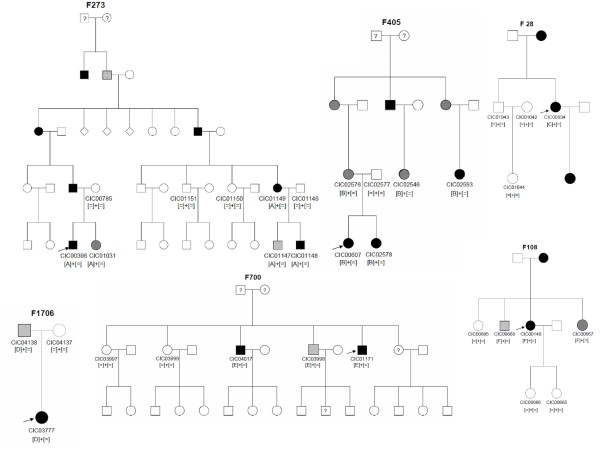
**Pedigrees of adRP patients with *PRPF31 *mutations and co-segregation in available family members**. Filled symbols represent affected, unfilled unaffected and dotted asymptomatic individuals. Question marks indicate that it is not clear whether the individual is affected or not. Squares depict males, circles females. Arrows mark the index patients. Equation symbols represent unaffected alleles. The identified mutations were abbreviated as followed: A = c.269_273del, B = c.527+2T>C, C = c.666dup, D = c.709_734dup, E = c.873_897dup and F = c.997delG.

In the current study we report the identification of novel *PRPF31 *mutations in six of the 90 adRP index patients. In total two deletions, three duplications and a splice site mutation all over the gene were identified (Table 2). The respective deletions and duplications were predicted to lead to premature stop codons. The novel splice site mutation c.527 + 2T>C resides in the highly conserved donor site of exon 6, which is predicted to lead to skipping of exon 6. Co-segregation analysis revealed in all but one family incomplete penetrance (Table 2, Figure 1).

**Table 2:** Novel *PRPF31 *mutations in a French adRP cohort.

Index (families)	Exon	Nucleotide Exchange	Protein Effect	Information about Penetrance
CIC00398 (F273)	4	c.269_273del	p.Tyr90CysfsX21	incomplete

CIC00607 (F405)	Int6	c.527+2T>C	splice defect	incomplete

CIC00034 (F28)	7	c.666dup	p.Ile223TyrX56	segregates (1 affected show mutation, 3 unaffected no mutation)

CIC03777 (F1706)	8	c.709_734dup	p.Cys247X	incomplete

CIC01171 (F700)	9	c. 873_897dup	p.Thr300GlyfsX32	incomplete

CIC00140 (F108)	10	c.997delG	p.Glu333SerfsX5	incomplete

Mutations are indicated according to NM_015629.3 by using the recommendations of human genome variation society: http://www.hgvs.org/rec.html.

To detect large deletions in those patients (60 patients) where no mutations was detected by direct sequencing approaches, MLPA studies were performed. However, no additional deletion was found by this method.

**Figure 2:**
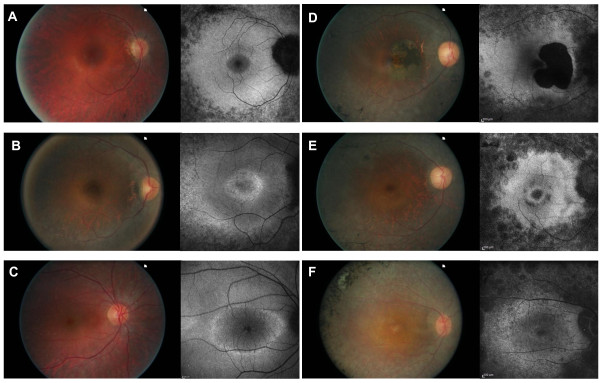
**Color fundus photograph and autofluorescence imaging of the right eye for each index patient: A: CIC0398; B: CIC00607; C: CIC00034; D: CIC01171; E CIC00140; F: CIC03777**.

Phenotypic characteristics of the 6 index patients are summarized in table 3 and 4. Four of them were females and two males with age ranging from 23 to 44 years with an average age of 34.5 years. Age at time of diagnosis ranged from 5 to 43 with an average of 19.5 years. Symptoms that led to the diagnosis were dominated by night blindness in all patients but one (CIC01171). Two patients also complained of visual field constrictions (CIC00034 and CIC01171). Refractive errors were variable. Central visual acuity was relatively preserved except in one patient (CIC00140), age 31, who had decreased central vision that just qualified her for legal blindness. Preserved central vision was well correlated with preserved responses to central hexagons in multifocal ERG. All patients showed visual field constriction with some peripheral perception, except for one patient (CIC00607), age 23, who had a relatively normal binocular visual field. This patient was the only one with detectable responses for both scotopic and photopic condition on ERG. Color vision was normal in 3 patients or showed tritan defect in one eye for one patient (CIC00607) and both eyes for patients CIC00140 and CIC03777 who also had low visual acuity. Anterior segment examination showed moderate posterior subcapsular cataract only in one patient (CIC00034), age 42. Fundus examination showed typical peripheral signs of RP with variable posterior pole involvements (Figure 2) including one patient with cystoid macular edema (CIC00607, Figure 2B), one patient with an area of parafoveal well-demarcated atrophy (CIC00034, Figure 2C), two patients with perifoveal atrophic changes (CIC01171, Figure 2D, CIC03777, Figure 2F) and one patient with foveal thinning (CIC00140, Figure 2E). This would suggest that central involvement is not uncommon in the course of the disorders and that central changes can occur as early as age 31 (patient CIC00140, Figure 2E). These cone dysfunction and macular changes can lead to further decrease in central vision and central cone survival should be the major target of future therapeutic intervention.

**Table 3:** Clinical data of affected members from families with adRP due to *PRPF31 *mutations

Family and PRPF31 mutation	Patient	Age at time of testing	Age at time of diagnosis	Sex	Family history	Symptoms at time of diagnosis	BCVA OD/OS Refraction	Lens	Fundus examination	OCT	FAF
**F273**	**CIC398**	23	5	M	From North of Brittany Father affected and few	Night blindness	20/25 20/20 -6.25(-1.75)20° -5.50(-1.25)175°	clear	Normal disc color, narrowed retinal vessels, little RPE changes in the periphery,	Preserved foveal lamination	Loss of AF outside the vascular arcades, perifoveal ring of increased AF

**F405**	**CIC00607**	23	20	F	One sister affected, cousins on maternal side affected Mother not affected incomplete penetrance From French descent	Night blindness late teens	20/32 20/32 +0.75(-2.75)15° Plano(-1.75)175°	clear	Bilateral ERM Normal disc color; no narrowing of blood vessels; little changes in the periphery with few bone spicules	Bilateral ERM Bilateral CME	Perifoveal ring of increased AF; foveal changes due to CME

**F28**	**CIC00034**	42	18	F	Family from Cameroun, daughter mother and one brother affected, no notion of incomplete penetrance	Night blindness and visual field constriction	20/32 20/32 -3.25(-0.75)65° -3.25(-0.75)130°	Small posterior subcapsular opacities	Disc pallor narrowed, blood vessels, RPE changes in periphery, bilateral atrophic lesion off the fovea	Preserved foveal lamination	Loss of AF outside the vascular arcades, round eccentric parafoveal area of loss of AF, no ring of AF

**F1706**	**CIC03777**	44	8	F	Paternal grand-mother, great-grand mother and one great-uncle on father side affected, French family from Jewish Ashkenazi ancestry	Night blindness since age 7	20/125 20/160 +0.5(-1.25)40° +0.25(-1.50)155°	pseudophakic	Pale optic disc, narrowed retinal vessels	Preserved foveal lamination	Loss of AF outside the vascular arcades, patchy loss of AF within the posterior pole with no ring of AF

**F700**	**CIC01171**	44	43	M	One elder brother affected, one niece affected from one of his unaffected sister, one uncle on father side Incomplete penetrance family originating from the Mauritius Island	Visual field constriction, no real night blindness	20/32 20/25 -0.50(-3.75)15° -0.25(-3.75)175°	clear	Normal disc color, narrowed retinal vessels, RPE changes in the periphery	Preserved foveal lamination	Loss of AF outside the vascular arcades; small perifoveal ring of increased autofluorescence with some perifoveal areas of loss of AF

**F108**	**CIC00140**	31	23	F	Mother, maternal grand-mother affected; family from Ivory Coast	Night blindness since birth	20/500 20/200 +1.50(-1.25)95° +1.50(-1.25)75°	clear	No pale optic disc; narrowed retinal vessels, some RPE changes in the periphery	Foveal thinning	Loss of AF outside the vascular arcades, increased AF within the foveal region associated with some patchy loss of AF

BCVA Best Corrected Visual Acuity; CME: Cystoid Macular Edema; ERM: Epi Retinal Membrane; AF: autofluorescence; OCT: optic coherence tomography OD: Oculis dextra (right eye); OS: Oculis Sinistra (center eye); RPE: Retinal Pigment Epithelium.

**Table 4:** Functional data.

Patient	Color vision	Binocular Goldman visual field, III4 isopter	Full field ERG	Multifocal ERG
**CIC00398**	ODS normal at 28 saturated Farnworth Hue	20° both horizontally and vertically with a large island of perception in temporal and inferior periphery	Only residual cone responses	Relatively well preserved central responses

**CIC00607**	OD tritan defect; OS normal at Farnsworth 15Desaturated Hue	180° horizontally ×110° vertically	Rod-cone dysfunction with 80% of normal for scotopic 3.0cd.s/m^2 ^ERG amplitude and 50% of normal for photopic 3.0cd.s.m^2^	Relatively well preserved central responses

**CIC00034**	ODS normal at 28 saturated Farnworth Hue	20° both horizontally and vertically with 2 bitemporal island of perception in periphery	ND	Only preservation of responses to central hexagons

**CIC03777**	ODS tritan defext at 28 saturated Farnworth Hue	20° both horizontally and vertically	ND	Only residual responses to central hexagons

**CIC01171**	ODS normal at 28 saturated Farnworth Hue	20° both horizontally and vertically	ND	Only preservation of responses to central hexagons

**CIC00140**	ODS tritan defext at 28 saturated Farnworth Hue	30° both horizontally and vertically with a large island of perception in temporal and inferior periphery	ND	Only residual responses to central hexagons

NP: not performed; ND: not detectable, OD: Oculus Dexter; OS: Oculus Sinister

Due to the small number of index patients included in this study, it is difficult to draw general conclusions on phenotypic variability and phenotype/genotype correlation. However, our cohort still shows on one hand one patient with reduced but still detectable rod-cone responses and well preserved central vision (CIC00607) at age 23 and on the other hand one legally blind patient with undetectable ERG (CIC00140), at age 31, suggesting variable severity of the disorder. This variable severity of the disorder is in accordance with previous reports, which also mentioned the possible role of unknown modifier genes [15]. Further longitudinal studies are required to document retinal degeneration kinetics and especially macular involvement in order to prepare the patients for future treatment.

With the study presented here we report 6 novel mutations in a French cohort leading to variable severity of adRP. To our knowledge, this is the first report on *PRPF31 *mutation screening and prevalence in the French population and it further expands the mutation spectrum causing adRP. In total two deletions, three duplications and a splice site mutation were identified (Table 2). To date only few *PRPF31 *variations have been reported to be recurrent (Table 1). This holds also true for our study. Consistent with previous reports, we propose that these mutations also lead to loss of function of PRFP31 and thus to haploinsufficiency [19-21]. In five of our families incomplete penetrance was observed. Only in one family (family 28) no asymptomatic mutation/or obligate carriers were reported. This was confirmed on three unaffected family members who did not reveal a mutation. However, due to the small size of the family, incomplete penetrance cannot be formally excluded for this mutation. To our knowledge, to date only one large Chinese family was reported with high penetrance [22], suggesting that most of the *PRPF31 *mutations are indeed associated with incomplete penetrance. Although the presumed mechanism to explain this phenomenon is allelic imbalance with over-expression of the wild-type allele, compensating for the non-functional allele in asymptomatic carriers [21,23], the exact mechanism how this over-expression happens remains to be solved.

Patient data and mouse *in vivo *studies strongly suggest that the disease mechanism is caused by haploinsufficiency rather than dominant negative effect. A recent study in mice demonstrated that p.A216P mutation as well as deletion of *Prpf31 *exon 7 in mice lead to null alleles [24]. Mice heterozygous for these mutations did not reveal signs of retinal degeneration in histological, ERG and fundus examination, however in homozygous state they were embryonic lethal, demonstrating lack of function of the mutant *Prpf31 *alleles. In a number of studies a cytotoxic effect of *PRPF31 *mutations has been suggested [25,26]. We believe that this toxicity plays a minor role in the development of the disease since asymptomatic mutation carriers do not develop retinal degeneration.

The study presented here reveals a prevalence of 6.7% in adRP cases due to *PRPF31 *mutations. This is higher than in another study from UK with 5% [15], in a study from India (4%), from Japan with 3% [12], from Spain with 1.7% [11] and from China with 1% [10]. A prevalence study by Sullivan and co-workers (2006) in 200 US families of presumably UK origin revealed 5.5% of cases with *PRPF31 *mutations. However, these numbers were corrected to 8% when MLPA studies revealed larger deletions, which were not detectable by direct sequencing approaches [14]. In contrast to these findings, our MLPA studies did not reveal any large deletions or duplications in this cohort. Therefore, we conclude that genomic rearrangements in the *PRPF31 *gene are not common in the French adRP cohort.

## Conclusions

With the study presented here we report six novel mutations in a French cohort leading to variable severity of adRP in families with mainly incomplete penetrance. In 6.7% of this cohort *PRPF31 *mutations were detected, rendering this gene a major gene for adRP in France. Consistent with previous reports, we propose that mutations in *PRPF31 *are mainly not recurrent, lead to loss of function of PRFP31 and thus to haploinsufficiency.

## Competing interests

The authors declare that they have no competing interests.

## Authors' contributions

IA contributed to the design of the study, the acquisition and interpretation of clinical data, and drafted the manuscript. KB contributed to the design and interpretation of the MLPA studies. S MS contributed to the acquisition and interpretation of clinical data. M-E L, V M-D, NH W and A A performed the DNA extraction and sequence analysis. J-A S contributed to the design of the study. SSB contributed to the design of the study, and helped to draft the manuscript. CZ contributed to the design of the study, the acquisition and interpretation of molecular genetic data, and drafted the manuscript. All authors read and approved the final manuscript.

## Pre-publication history

The pre-publication history for this paper can be accessed here:

http://www.biomedcentral.com/1471-2350/11/145/prepub
